# A Selective Separation Mechanism for Mono/divalent Cations and Properties of a Hollow-Fiber Composite Nanofiltration Membrane Having a Positively Charged Surface

**DOI:** 10.3390/membranes14010001

**Published:** 2023-12-20

**Authors:** Enlin Wang, Xinghua Lv, Shaoxiao Liu, Qiang Dong, Jiayue Li, Honghai Li, Baowei Su

**Affiliations:** 1 Key Laboratory of Marine Chemistry Theory and Technology, Ministry of Education/College of Chemistry & Chemical Engineering, Ocean University of China, 238 Songling Road, Qingdao 266100, China; wangenlin_ouc@126.com (E.W.); oucchemlxh@163.com (X.L.); lsx158799@foxmail.com (S.L.); dongqiang_ouc@126.com (Q.D.); lee_ouc@126.com (J.L.); 2 College of Chemical Engineering, Qingdao University of Science and Technology, Qingdao 266045, China; lihonghai@qust.edu.cn

**Keywords:** nanofiltration, hollow-fiber membrane, positively charged, interfacial polymerization, mono/divalent cation separation

## Abstract

Positively charged nanofiltration (NF) technology is considered a green and low-cost method for mono/divalent cation separation. Nevertheless, the separation rejection mechanisms of these NF membranes have yet to be extensively investigated. In this work, we fabricated a thin-film composite (TFC) hollow-fiber (HF) NF membrane with a positively charged surface via modification of the nascent interfacial polymerization layer using a branched polyethyleneimine (BPEI)/ethanol solution. Then, we extensively investigated its selective separation mechanism for mono/divalent cations. We proposed and proved that there exists a double-charged layer near the membrane surface, which helps to repel the divalent cations selectively via Donnan exclusion while promoting the fast penetration of monovalent cations. Meanwhile, the membrane skin layer is loose and hydrophilic due to the loose BPEI structure and the abundance of amine groups, as well as the changed fabrication conditions. In this way, we achieved very good mono/divalent cation selectivity and relatively high water permeance for the as-prepared HF NF membrane. We also obtained good anti-fouling, anti-scaling, and acid resistance, and long-term stability as well, which are urgently needed during practical application. Furthermore, we successfully amplified this HF NF membrane and proved that it has broad application prospects in mono/divalent cation separation.

## 1. Introduction

Mono/divalent cation separation involves important chemical processes such as energy conversion and storage, environmental pollution detection, clean industrial production, and resource recovery and reuse, especially in the fields of salt-lake lithium extraction, brine refining, high-salt wastewater recycling, and so on. For example, it has recently become a research focus to extract lithium, the highly valuable energy metal, from salt-lake brine, since nearly 70% of lithium resources are located in salt lakes around the world [1,2,3], but there exists great difficulty due to the very high mass ratio of Mg^2+^/Li^+^ therein and the similar hydrated ionic radii of these two kinds of ions [4]. Positively charged nanofiltration (NF) membranes are efficient and low-cost for mono/divalent cation separation, as they have excellent selectivity for divalent cations over monovalent cations [5,6,7] via Donnan exclusion and size sieving [8,9]. Recently, the fabrication of these membranes has become a research hotspot, and the most commonly used methods are interfacial polymerization [10,11] and surface modification [12,13].

For positively charged membranes fabricated via interfacial polymerization, polyethyleneimine (PEI) or a mixture of PEI and piperazine (PIP) is usually used as the aqueous phase monomer [14,15,16,17] to react with TMC in *n*-hexane. For example, Fang et al. [16] successfully prepared a kind of branched PEI (BPEI)/TMC interfacial polymerization hollow-fiber (HF) NF membrane and achieved a MgCl_2_ rejection of 96.7% and a moderately high NaCl rejection of 42%, as well as a remarkably high pure-water permeability (PWP) of 171 L m^−2^ h^−1^ MPa^−1^ due to the greater free volume that the branched structure of BPEI molecules provides for water permeation, which demonstrates that the use of BPEI as the aqueous monomer or mixed aqueous monomer could achieve a reasonably high mono/divalent cation selectivity. Fang et al. [17] also proved that using PIP solely as an aqueous monomer could greatly decrease NaCl rejection while keeping the high MgCl_2_ rejection unchanged. This gives us a good indication that PIP might be a suitable candidate as an aqueous monomer for increasing the rejection difference between multi- and monovalent cations.

Apart from PEI and PIP as aqueous monomers, researchers [18,19] have also used other chemicals as monomers during interfacial polymerization. For example, recently, Peng et al. [18] synthesized a quaternized-spiral piperazine (QSPIP) featuring a spiral conformation and quaternary/secondary amines and used it as an aqueous monomer to react with TMC via interfacial polymerization and fabricated a kind of QSPIP-TMC membrane, which achieved an approximately five-times-improved water permeance (220 LMH/MPa) compared with analogous polyamide membranes and a good Mg/Li selectivity of 8.7. This work provides a new idea for mono/divalent cation separation.

However, during the interfacial polymerization process, the abundance of residual acyl chloride groups would hydrolyze to carboxyl groups [20] and neutralize the membrane surface positive charge, thereby reducing mono/divalent cation selectivity [21,22]. Many researchers [7,18,23,24,25] have carried out surface modification after the interfacial polymerization to improve the surface positive charge density. For example, Peng et al. [18] and Liu et al. [25] synthesized positively charged ionic liquid monomers to modify nascent flat-sheet PEI/TMC polyamide TFC membranes and achieved a reasonably high PWP of over 200 L m^−2^ h^−1^ MPa^−1^ and a very high MgCl_2_ rejection (about 95%) with a very low LiCl rejection (34%) for their NF membrane and a satisfactory *S*_Li/Mg_ of higher than 10. This is a good routine to obtain positively charged NF membranes with an excellent selectivity as well as a high water permeance. Lu et al. [26], from another perspective, used PEI in ethanol to modify the surface of a nascent flat-sheet PIP/TMC polyamide layer and obtained a satisfactory *S*_Li/Mg_ of higher than 12 at a mass ratio of 150 for Mg^2+^/Li^+^ with a water permeance of nearly 90 L m^−2^ h^−1^ MPa^−1^. This indicates to us that using ethanol as the modification solvent could inhibit acyl chloride hydrolysis and could increase the difference in rejection between multi- and monovalent cations. However, until now, most research works have focused on the surface modification of flat-sheet NF membranes. Recently, researchers [27,28] have also carried out the surface modification of hollow-fiber NF membranes. For example, Yin et al. [27] used aqueous tris(2-aminoethyl) amine solution to modify the polyamide layer and achieved a very high MgCl_2_ rejection of 97.6% and a reasonably high PWP of 160 L m^−2^ h^−1^ MPa^−1^. Their work gives us a good indication for fabricating HF NF membranes with a positively charged surface and high divalent cation rejection together with a high water permeance.

Nevertheless, for positively charged NF membranes, when the rejection of MgCl_2_ is higher than 95%, LiCl or NaCl rejection is also usually relatively high, such as higher than 40%, thus resulting in relatively low mono/divalent cation selectivity. Elemelech et al. [29] considered that, for desalination membranes, the critical thing is to increase selectivity. We consider that the same is true for positively charged NF membranes. The relatively low mono/divalent cation selectivity could be due to either the high charge density of the skin layer or to the narrow membrane pores. From the relatively high PWP, we could deduce that the pore diameter might not be so small. Therefore, the key factor that influences mono/divalent cation selectivity might be the charge density of the skin layer.

Recently, we carried out a preliminary work based on the surface modification of an internally pressured positively charged HF NF membrane using interfacial polymerization between PIP and TMC followed by BPEI modification in ethanol [30] and obtained a good result for Mg/Li selectivity. However, the effect of membrane charge structure on the mechanism of cation rejection has not yet been investigated in detail. We believe that if the polyamide layer matrix is less positively charged, monovalent caions will more easily penetrate the polyamide layer after diffusing through the surface modification layer. However, until now, no extensive research has been carried out on this subject. Therefore, in this work, we extensively analyzed the effects of surface modification with BPEI in ethanol on the surface chemistry and the charge distribution along the vertical direction of the polyamide layer matrix as well as on the performance of an internally pressured positively charged HF NF membrane. Firstly, we propose a double-charged-layer concept and extensively investigate and demonstrate the mechanism of inhibiting the residual acyl chloride groups from hydrolyzing by using ethanol as the solvent during the surface modification and the mechanism of the subsequent shielding of the negative charge density on the polyamide layer surface. Then, we evaluate its surface physio-chemical properties, separation, and stability performance, including its anti-scaling and anti-fouling performance. Finally, we amplify the internally pressured positively charged HF NF membrane module. Our work has great implications for the design and development of positively charged HF NF membranes.

## 2. Materials and Methods

### 2.1. Materials

Piperazine (PIP), trimesoyl chloride (TMC), polyvinylpyrrolidone (PVP), bovine serum albumin (BSA), branched polyvinylimide-800 (BPEI, average Mw of ~800 by Light scattering method), and polyethylene glycols (PEGs, 200, 400, 600, and 1000 Da) were obtained from Shanghai Maclin Biochemical Technology Co., Ltd. (Shanghai, China). Polysulfone (PSf) power was purchased from Solvay Corp., Belgium. *N*,*N*-dimethylformamide (DMF) and *n*-hexane were purchased from Tianjin Fuyu Fine Chemical Co., Ltd. (Tianjin, China). Anhydrous ethanol (EtOH), NaCl, NaOH, NaClO, Na_2_SO_4_, LiCl, and MgCl_2_ were purchased from Sinopharm Chemical Reagent Co., Ltd. (Shanghai, China).

### 2.2. Fabrication of the HF Membranes

**Figure 1:**
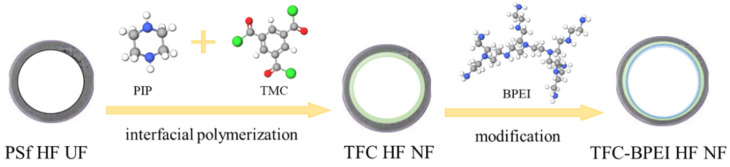
The preparation diagram of the HF NF membrane.

HF ultrafiltration (UF) substrates were fabricated using PSf power according to the literature [31,32], using the specific membrane preparation parameters listed in the Supplementary Materials S1. A TFC HF NF membrane with a positively charged surface was fabricated according to our recent work [30], and was termed TFC-BPEI-*x* (TFC-BPEI for short), in which *x* represents the BPEI concentration in the ethanol solution. The preparation process is presented in Figure 1. The BPEI surface modification conditions are presented in Table 1. For comparison of the surface modification effects, the membrane before BPEI modification is termed TFC-0.

### 2.3. Characterizations

X-ray photoelectron spectroscopy (XPS), scanning electron microscopy (SEM), atomic force microscopy (AFM), water contact angle (WCA) goniometer, and Zeta potential measurements were used to characterize the membrane surface’s physical–chemical properties. The details of the characterized instruments, such as for SEM, FTIR, and AFM, are presented in Supplementary Materials S2 and Supplementary Materials S3.

### 2.4. Separation Performance

The separation performance tests were performed under 0.4 MPa and 25 °C with a crossflow filtration platform without specification. More than three HF NF modules under each fabrication condition were tested to ensure reproducibility. The separation performance is expressed in term of water permeance (*P*) and solute rejection (*R*) as in our previous work [30]. MgCl_2_ and LiCl single-salt solutions with different concentrations were used to test the concentration effect. A 2000 mg L^−1^ aqueous mixture with mass ratios of 20, 50, 100, and 200 for Mg^2+^/Li^+^ was used to evaluate the membranes’ selectivity. The ion concentrations were measured with ion chromatography (Shimadzu, Kyoto, Japan) and the separation factor for Mg^2+^/Li^+^ is expressed as Equation (1). The separation factor for ions measured using single-salt solutions can also be calculated using this equation.

(1) $$S_{N/M}=\frac{1-R_{N}}{1-R_{M}}$$

where subscript N means monovalent ions in the mixed-salt solution or the single-salt solution having monovalent cations and subscript M means divalent cations in the mixed-salt solution or the single-salt solution having divalent cations. 

The feasibility of extracting and concentrating LiCl from the MgCl_2_ solution with the fabricated HF NF membranes was tested using a two-pass HF NF separation process. By measuring the ion concentration in the permeate of the first NF process and the secondary NF process, the separation factors for both separation processes are calculated using Equation (1).

The ion permeation rate was evaluated using an ion diffusion experiment, for which the operation process is presented in Supplementary Materials S4. Briefly, 0.2 mol L^−1^ NaCl, LiCl, and MgCl_2_ solutions were, respectively, pumped into the lumen of the HF NF membrane, and DI water was pumped into the shell side. Then, ions diffuse to the shell side by the driving force of the concentration difference. The permeation rate of the ions (*P*_i_) is calculated by measuring the change in the conductivity of the shell-side solution with time using Equation (2).

(2) $$P_{i}=\frac{C_{i}\cdot V}{A\cdot t}$$

where *C*_i_ means the cation molecular concentration in the permeate solution, *V* is the volume of the permeate solution, *A* is the effective membrane area, and *t* is the diffusion time interval. 

For the fabricated HF NF membranes, the molecular weight cut-offs (MWCO) were evaluated using 2000 mg L^−1^ PEG solutions of different molecular weights. The pore size distribution of the HF NF membrane surface was evaluated according to the literature [33,34], using the following log-normal distribution model, Equation (3) [33], based on the PEG rejection data.

(3) $$\frac{dR\left(d_{p}\right)}{dd_{p}}=\frac{1}{d_{p}ln\sigma_{p}\sqrt{2\pi }}exp\left[-\frac{{\left(lnd_{p}-ln\mu_{p}\right)}^{2}}{2{\left(ln\sigma_{p}\right)}^{2}}\right]$$

where the average molecular Einstein–Stokes diameter (*μ*_p_) and standard deviation (*σ*_p_) as well as the Einstein–Stokes diameter (*d*s) of the PEGs is calculated according to Prof. Chung et al. [33].

### 2.5. Stability Evaluation

All the stability tests were performed using the crossflow filtration platform under the condition of 25 °C and 0.4 MPa. 

The scaling resistance of the membranes was investigated according to the literature [26]. First, the initial flux (*J*_0_) of the membranes was measured with an aqueous solution consisting of 120 mM NaCl and 1 mM CaCl_2_ (ionic strength 0.12 M) as the feed solution. Then, the feed solution was replaced with another aqueous mixture with the same initial ionic strength but containing 21 mM NaCl, 19 mM Na_2_SO_4_, and 23 mM CaCl_2_. Under the same test condition, the flux (*J*_t_) of the membrane was recorded for 12 h, and the scaling resistance of the membrane is expressed as the normalized flux (*J*_N_), which is shown in Equation (4).

(4) $$J_{N}=\frac{J_{t}}{J_{C}}$$

After the test, the membrane modules were cleaned for 30 min. Then, their water flux (*J*_C_) was tested again under the same conditions using a solution containing 120 mM NaCl and 1 mM CaCl_2_ as feed, and the flux recovery rate of the membrane was calculated using Equation (4).

The antifouling performance was evaluated using a 500 mg L^−1^ BSA solution. Firstly, ultra-pure water was pumped into the membrane modules for 1 h. Afterward, BSA solution was pumped into the membrane modules for 1 h. Finally, ultra-pure water was used again as feed for 1 h. The water flux rates in the three periods were measured every 10 min and were termed the initial water flux (*J*_0_), the water flux in the fouling solution (*J*_t_), and the water flux after cleaning (*J*_C_), respectively. The antifouling performance was characterized in terms of both water flux decline rate, (*FDR*) as shown in Equation (5),

(5) $$FDR=\left(1-\frac{J_{t}}{J_{0}}\right)\times 100\%$$

and water flux recovery rate (*FRR*) (normalized flux (*J*_N_)), which is calculated using Equation (4) using the ratio of *J*_C_ and *J*_0_.

Chemical stability was evaluated by immersing the HF NF membranes in HCl solution. At first, initial water flux (*J*_0_) and salt rejection (*R*_0_) were tested using 2000 mg L^−1^ MgCl_2_ solution. Then, the membranes were run at low pressure for 5 d with a hydrochloric acid solution of pH = 3 as the feed solution. During the cycling period, the membranes were rinsed with deionized water every 24 h, then water flux (*J*_t_) and salt rejection (*R*_t_) were measured under the same conditions. The normalized flux (*J*_N_) was calculated from Equation (4) and the normalized salt rejection (*NSR*) was calculated from Equation (6). These data reflect the decay of the flux and rejection.

(6) $$NSR=\frac{R_{t}}{R_{0}}$$

Three modules were run for 40 h. The separation performance was tested every 2 h. The separation performance was averaged with respect to the operating time.

## 3. Results and Discussion

### 3.1. Optimization of the Surface Modification Conditions

**Figure 2:**
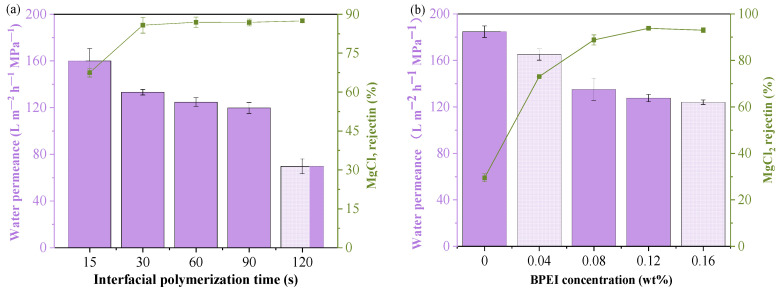
Effects of interfacial polymerization time with a BPEI of 0.08 wt% in ethanol and a surface modification time of 2 min (**a**) and of BPEI concentration (**b**) on membrane separation performance. Fixed aqueous PIP concentration of 0.2 wt%, aqueous immersion time of 3 min, and organic TMC concentration of 0.15 wt%.

The conditions for fabricating the TFC-0 membrane included 0.2 wt% PIP, 0.15 wt% TMC, and an interfacial polymerization time of 90 s, as these conditions could achieve the optimal separation performance for the TFC-0 membrane. However, when the surface of this membrane was modified by the BPEI ethanol solution, these optimal fabrication conditions were not suitable for improving the separation performance of TFC-BPEI. Therefore, the effect of interfacial polymerization time at a fixed BPEI concentration of 0.08 wt% in ethanol was investigated at a fixed surface modification time of 2 min, as shown in Figure 2a. The effects of BPEI concentration on the separation performance of the HF NF membranes are shown in Figure 2b.

With the interfacial polymerization time extended from 15 s to 30 s, the water permeance gradually drops from 160.0 to 124.6 L m^−2^ h^−1^ MPa^−1^, accompanied by a vigorously increased MgCl_2_ rejection from 67.5% up to 85.8%. Compared with the water permeance of TFC-0 at 30 s, that of the BPEI-modified membrane increases, demonstrating that the BPEI modification is also beneficial for water permeation. With further prolonging of the interfacial polymerization time, MgCl_2_ rejection does not change significantly. This indicates that for the BPEI-modified positively charged HF NF membrane, the polyamide layer could achieve sufficient crosslinking at an interfacial polymerization time of 30 s. Further extension of the interfacial polymerization time would result in a much denser and thicker polyamide layer, which is disadvantageous for water permeation. That is, the optimal interfacial polymerization conditions used for TFC-BPEI cannot be the same as those of TFC-0. According to the solute diffusion model and pore flow model [35], both the polyamide layer and the BPEI modification layer would have an effect on the water permeation. The polyamide layer mainly influences the water flux, and the BPEI modification layer mainly influences the surface positive charge and the selectivity for cations. With the synergistic effects of the BPEI modification layer and the polyamide layer, at the shorter interfacial polymerization time, the hydraulic resistance of the polyamide layer could decrease significantly while not sacrificing divalent cation rejection. That is, the secondary reaction could not only realize regulation of the charge property of the polyamide layer and increase its separation selectivity for divalent cations, but also greatly shorten the interfacial polymerization time, which, in turn, could result in a thinner polyamide skin layer. Therefore, the formed polyamide layer of TFC-BPEI is looser and thinner, and the optimal interfacial polymerization time for TFC-BPEI was selected as 30 s.

However, as the BPEI concentration used in the above investigation was 0.08 wt%, the BPEI-modified HF NF membrane still had relatively low rejection for divalent cations. Therefore, the effect of BPEI concentration was further investigated, as presented in Figure 2b. We used an aqueous PIP concentration of 0.2 wt%, aqueous contact time of 3 min, organic TMC concentration of 0.15 wt%, interfacial polymerization time of 30 s, BPEI modification time of 2 min, and ultra-pure water flushing time of 5 s. The MgCl_2_ rejection increased from 29.5% to 93.8% with the BPEI concentration increasing from 0 wt% to 0.12 wt%, which indicates that BPEI modification can bring enough positive charge for divalent cation rejection. However, as the BPEI concentration further increased to higher than 0.12 wt%, MgCl_2_ rejection and water permeance remained basically unchanged, as the limited residual unreacted acyl chloride groups could be easily used up at BPEI concentrations higher than 0.12 wt%. At the same time, due to the macromolecular segment structure and hydrophilic characteristics of BPEI, a BPEI-modified HF NF membrane must have smooth morphology and increased hydrophilicity [3,12]. Therefore, the optimal concentration of BPEI is 0.12 wt%, and the optimal HF NF membrane is termed TFC-BPEI-0.12, abbreviated as TFC-BPEI for short in the following sections.

### 3.2. Characterization of the Membranes’ Surface

We investigated the mechanism of selective separation in TFC-BPEI using XPS, together with the aid of ATR-FTIR, Zeta potential, hydrophilicity, as well as surface physico-chemical property characterizations.

#### 3.2.1. Surface Chemistry and Physico-Chemical Properties

**Figure 3:**
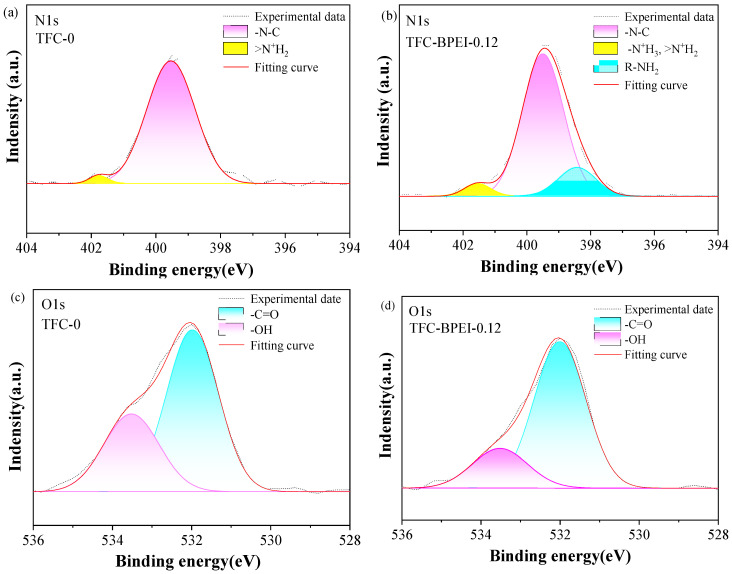
Devolution of N1s, O1s, and C1s XPS spectra for TFC-0 (**a**,**c**) and TFC-BPEI-0.12 (**b**,**d**).

The chemical circumferences of the membrane surfaces of the TFC-BPEI and TFC-0 membranes were extensively investigated by XPS. The wide-scan XPS spectra and the atomic percentages for both membranes are shown in Supplementary Materials S5. The O element has been detected on the surface of TFC-BPEI. As BPEI has no O element, the O element must come from the acyl groups of TMC. This means that the XPS detection must reach the poly(piperazine) amide layer, which is formed via interfacial polymerization between PIP and TMC. That is, the BPEI layer thickness is surely less than 10 nm, as the detection depth of XPS is usually about 10 nm. The devolution of the N1s and O1s XPS spectra for both membranes according to the literature [36] are presented in Figure 3. From the N1s peak fitting we can see that the peak area for the C-N groups is apparently larger for TFC-BPEI than for TFC-0, as compared in Figure 3a,b, which qualitatively indicates the successful grafting of the amine groups after BPEI modification. The emerging large peak of the R-NH_2_ groups from the N1s peak fitting of TFC-BPEI further qualitatively demonstrates the BPEI modification [36,37]. The O1s peak fitting also qualitatively demonstrates the BPEI modification. It can be seen that the peak area of the -OH groups is apparently smaller for TFC-BPEI than for TFC-0, as compared in Figure 3c,d. In the experimental conditions, the -OH groups must belong to the carboxyl groups attributed to the hydrolyzation of the residual unreacted -COCl groups. Therefore, this indicates that the negative charge density is much less on the surface of TFC-BPEI than on that of TFC-0.

The quantitatively evaluated contents of the chemical groups from the devolution of the N1s and O1s XPS spectra are listed in Table 2. This clearly shows the increased number of -N^+^H_3_ groups and the newly emerging R-NH_2_ groups for the N element, which quantitatively proves successful BPEI modification. The content of R-NH_2_ increases from 0% to 12.7% after modification, which indicates that the BPEI-modified membrane surface contains a large number of amino groups [14]. Meanwhile, the content of -OH bonds of the O element decrease from 32.1% to 16.2% and the content of -C=O bonds of the O element increases from 67.9% to 83.9% after BPEI modification. Both of these indicate that surface modification with a BPEI/ethanol solution effectively inhibits the acyl chloride groups from hydrolyzation [26] and thus helps to form more amide bonds between the -NH_2_ groups of BPEI and the -COCl groups.

**Figure 4:**
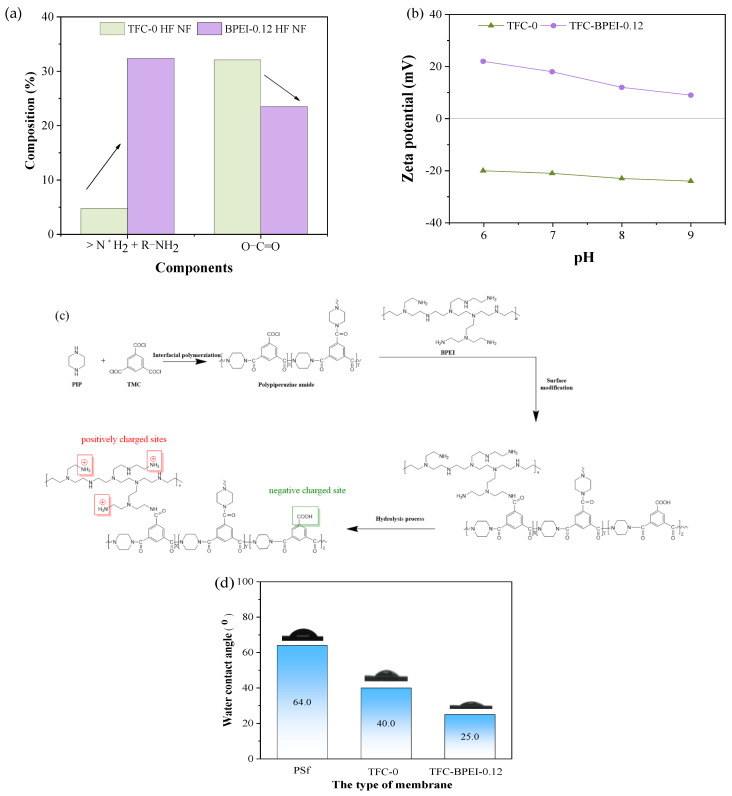
Changes in the content of ammonia groups and carboxyl groups in THC-0 and TFC-BPEI membranes (**a**), the Zeta potential of both TFC membranes (**b**), the reaction mechanism of TFC-BPEI (**c**), and the WCA values of the surface of the PSf substrate and the two TFC membranes (**d**).

In order to illustrate more intuitively the advantages of the modification step in this work, we plotted Figure 4a based on the data in Table 2. A much lower content of R-N^+^H_3_ and >N^+^H_2_ groups and a very high content of -OH groups are present on the TFC-0 surface. As explained above, this clearly indicates that TFC-0 has more carboxyl groups and is negatively charged. This has been further demonstrated by the Zeta potential measurement results. Under the test range of pH 3–9, the TFC-0 surface is negatively charged, as shown in Figure 4b, which is typical for PIP/TMC polyamide NF membranes. Comparatively, a very high content of R-NH_2_ and >N^+^H_2_/-N^+^H_3_ groups and a much lower content of -OH groups are detected on the surface of TFC-BPEI, as shown in Figure 4a. The total number of R-NH_2_ and >N^+^H_2_/-N^+^H_3_ groups increases from 2.4% to 17.1% after BPEI modification. This indicates that the BPEI-modified membrane surface contains a large number of amino groups, thereby realizing the charge reversal thereon [14]. In this way, the HF NF membrane shows a positively charged surface, as demonstrated in Figure 4b, indicating that post-interfacial polymerization modification using the BPEI/EtOH solution to react with local residual unreacted -COCl groups could achieve a high amino grafting degree.

Based on the above discussion, the surface modification mechanism of TFC-BPEI can be deduced and is presented in Figure 4c. During post-interfacial polymerization using the BPEI/ethanol solution, ethanol helps to inhibit the remaining -COCl groups on the pristine TFC-0 membrane surface from hydrolyzation. Therefore, more -COCl groups on the surface of the polyamide layer could react with the amine groups of the BPEI molecules to form amide groups and thus complete the grafting of a vast number of amine groups from the BPEI molecules. Only a small number of the acyl chloride groups remain unreacted during BPEI surface modification due to the steric hindrance effect and finally hydrolyze to carboxyl groups. At a neutral pH, the amine groups could easily absorb hydrogen ions to form positively charged ions [38]. In this way, the membrane surface has both negatively charged sites (-COO^−^) and positively charged sites (-N^+^H_3_). The number of the positively charged sites is much higher than that of the negatively charged sites due to the high content of amine groups on the BPEI chains. Therefore, the TFC-BPEI surface shows positively charged properties.

As both -NH_2_ and -COOH are hydrophilic groups, they could increase the membrane surface’s hydrophilicity. The contact angle measurement results in Figure 4d demonstrate that the water contact angle of TFC-0 decreases from 64.0° of the PSf HF UF membrane to 40.0°, since there are many hydrophilic -COOH and -NH- groups generated on the polyamide layer surface, as has been proved in Figure 3. Comparatively, the contact angle of TFC-BPEI further decreases to 25.0°, which indicates that its hydrophilicity is even better than that of TFC-0. Apparently, there are more R-N^+^H_3_ and R-NH_2_ groups on the surface of TFC-BPEI compared with those on TFC-0, as shown in Figure 4a, which indicates that the enhanced hydrophilicity of TFC-BPEI is caused by the more hydrophilic groups thereon due to the BPEI modification [39]. This, in turn, would be highly beneficial for enhancing the water permeance of TFC-BPEI.

#### 3.2.2. Surface Morphologies

**Figure 5:**
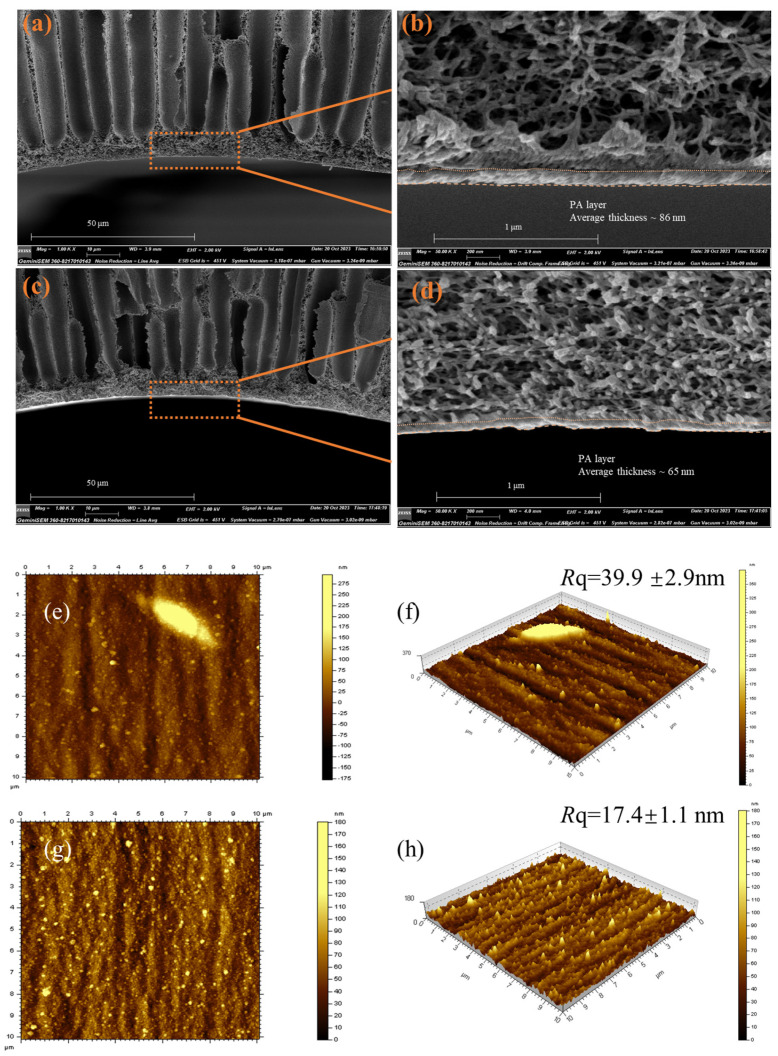
SEM image of two membranes, TFC-0 (**a**,**b**) and TFC-BPEI (**c**,**d**), and AFM images of the two TFC membranes, TFC-0 (**e**,**f**) and TFC-BPEI (**g**,**h**).

Figure 5a–d shows that the selective layers of TFC-0 and TFC-BPEI are about 86 nm and 65 nm respectively in thickness. This might be due to the optimization and shortening of the interfacial polymerization time of the TFC-BPEI membrane, which greatly reduces the number of organic phase monomers participating in interfacial polymerization, thereby reducing the thickness of the selective layer [40,41].

The surface AFM images of both membranes are presented in Figure 5e–h. There is a trough-like structure on the TFC-0 surface, with an average roughness of 39.9 nm. Compared with TFC-0, TFC-BPEI has a much smoother surface, with average roughness dropping sharply to 17.4 nm, which could be due to the deposition of BPEI to form a loose crosslinked BPEI layer and make the membrane surface smoother [12].

#### 3.2.3. Molecular Weight Cut-Off and Pore Size Distribution

**Figure 6:**
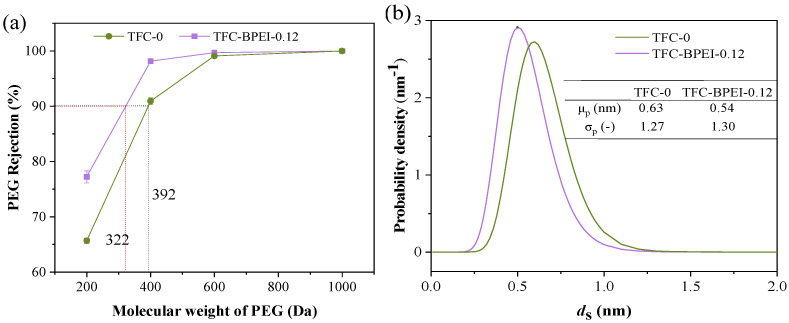
The rejection of PEG with different molecular weights (**a**) and the pore size distributions (**b**) of both TFC membranes.

The rejections of PEGs with different molecular weights for both TFC membranes are shown in Figure 6a. TFC-BPEI has a higher rejection than TFC-0 despite the different molecular weights used for PEG. According to the definition of molecular weight cut-off (MWCO), TFC-0 and TFC-BPEI have MWCOs of 392 Da and 322 Da, respectively. TFC-BPEI has a relatively lower MWCO, indicating that it has a more precisely selective separation ability. This could be attributed to the fact that BPEI modification reduces the average pore size of the polyamide skin layer.

As shown in Figure 6b, both membranes have similar geometric standard deviations (*σ*_p_), but TFC-BPEI has a smaller average pore diameter (*μ*_p_) than TFC-0 and has a higher probability density around the average pore diameter, which is highly beneficial for more precisely selective separation.

#### 3.2.4. In-Depth Charge Properties

**Figure 7:**
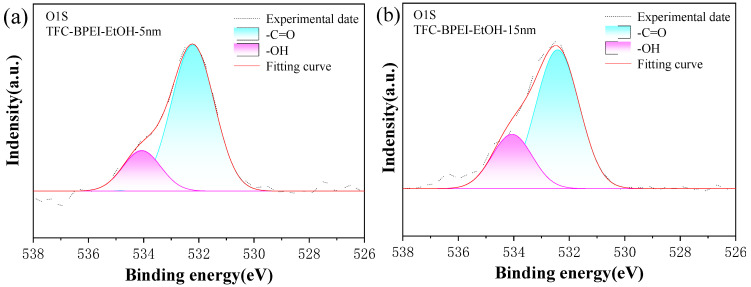

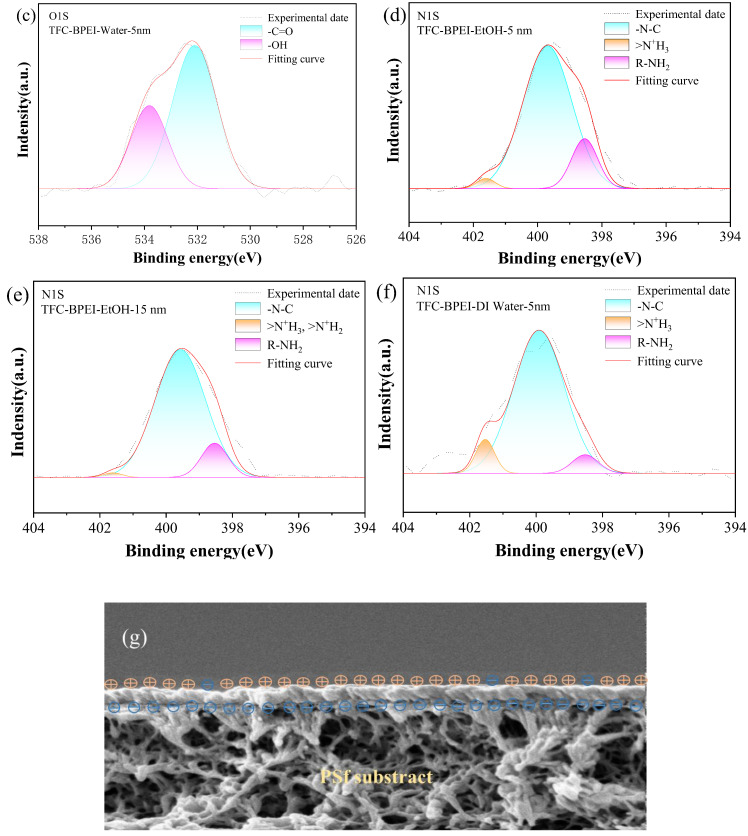
XPS N1s and O1S spectra for TFC-BPEI-EtOH-5nm, TFC-BPEI-EtOH-10nm, and TFC-BPEI-Water-5nm membranes (**a**–**f**); the double-charged structure of the modification layer (**g**).

It should be pointed out that as the XPS detection depth is about 10 nm, the above charge information in Section 3.2.1 just reflects the overall charge performance of the 10-nm-thick top skin layer. We speculate that the in-depth charge distribution will also influence the transmission of the cation ions. To further understand the variation in the charge properties with the depth of the skin layer, we performed XPS sputtering of the TFC-BPEI surface to depths of 5 nm and 15 nm, respectively, and measured the local chemical circumference; the corresponding XPS spectra are labeled TFC-BPEI-EtOH-5nm and TFC-BPEI-EtOH-15nm, respectively. The N1s and O1s peak fitting results are shown in Figure 7a–f and Table 3. For comparison, we also present the XPS fitting results for TFC-BPEI-Water-5nm.

The detection of the O element means that the XPS must reach the poly(piperazine) amide layer, as we mentioned in Section 3.2.1. Usually, during the modification process, BPEI molecules cannot react completely with all the residual acyl chloride groups on the nascent TFC-0 membrane surface, due to the high steric hindrance effect of the BPEI molecules. Hence, a small number of the residual acyl chloride groups will remain unreacted after BPEI/ethanol modification and subsequently hydrolyze to -COOH groups. For TFC-BPEI modified by a BPEI/ethanol solution, the percentage of -OH groups are about 19.6% at a sputtering depth of 5 nm, and the percentage of corresponding local -COOH groups is deduced to be about 40%, which means that 60% of the acryl groups have conducted the interfacial polymerization reaction to form amide groups thereon. Comparatively, the percentage of -OH groups is about 23.3% at a sputtering depth of 15 nm, and the corresponding percentage of local -COOH groups is deduced to be about 46%, which means that 54% of the acyl groups have conducted the interfacial polymerization reaction to form amide groups thereon. The increasing content of -OH groups with sputtering depth can be well explained by the residual acyl chloride groups hydrolyzing during the interfacial polymerization process, as the aqueous phase solution on the substrate surface contains plenty of water. A deeper sputtering depth means that the distance is shorter to the substrate surface, i.e., nearer to the aqueous phase, which indicates that the local acyl chloride groups can more easily hydrolyze. That is, carboxyl content gradually increases with the depth of the separation layer, which corresponds to the increase in the content of -OH in the XPS results. Therefore, the negative charge density increases with the depth of the poly(piperazine) amide layer. On the other hand, as the sputtering depth increases from 5 nm to 15 nm, the total content of -N^+^H_3_, >N^+^H_2_, and R-NH_2_ decreases from 17.4% to 13.3%, which indicates that the number of the positively charged sites gradually decreases. This corresponds to a decrease in the positive charge density of the separation layer along the depth direction.

To demonstrate the difference between ethanol and pure water as the solvent in the BPEI modification solution, we used 0.12 wt% BPEI/DI water to modify the inner surface of TFC-0, and the modified membrane is termed TFC-BPEI-Water. Then, we carried out XPS sputtering of the membrane surface to a depth of 5 nm, and the results are also shown in Figure 7 and Table 3. Comparing the spectra of TFC-BPEI-Water-5nm and TFC-BPEI-EtOH-5nm, we can see that the content of -C=O in the O1s spectrum of TFC-BPEI-Water-5nm is 32.5%, which is significantly higher than that of TFC-BPEI-EtOH-5nm (19.6%). This indicates that DI water as the BPEI modification solvent could exacerbate the hydrolyzation of the residual acyl chloride groups after the interfacial polymerization process. In contrast, the total content of -N^+^H_3_, >N^+^H_2_, and R-NH_2_ in the N1s spectrum of TFC-BPEI-Water-5nm is 13.2%, which is lower than that of TFC-BPEI-EtOH-5nm (17.4%). Therefore, under the same BPEI modification reaction parameters, the degree of amino grafting in TFC-BPEI-Water-5nm is lower than that of TFC-BPEI-EtOH-5nm, which means that the density of positive charges on the surface of TFC-BPEI-Water is also lower than that of TFC-BPEI-EtOH. Therefore, ethanol is better than water as the solvent for BPEI modification to obtain a more positively charged surface.

In summary, for the positively charged surface of the membrane in this work, the topmost BPEI modification layer is loose and positively charged, and the inner poly(piperazine) amide layer is relatively loose, very thin, and negatively charged. Thus, the charge distribution in the skin layer is just a double-charged layer, as illustrated in Figure 7h. This configuration is highly beneficial for monovalent cation transportation after these ions penetrate through the positively charged outer layer. 

### 3.3. Separation Performance

**Figure 8:**
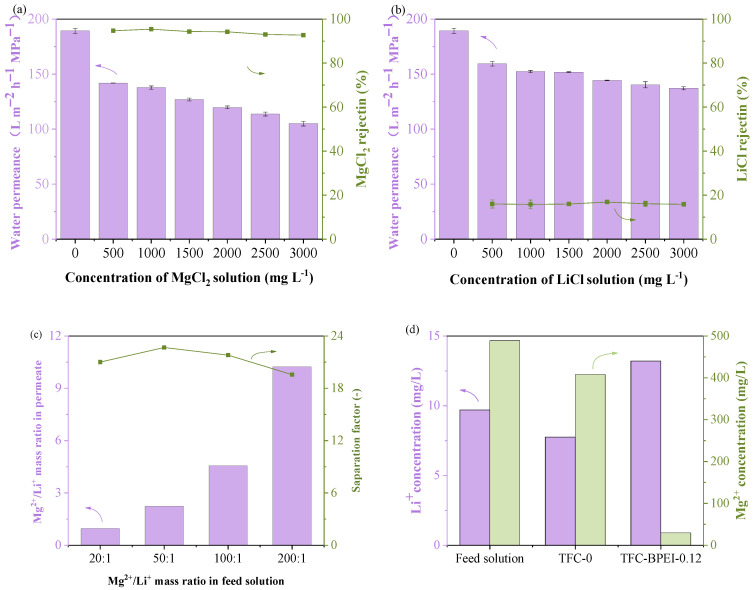
Effects of the concentrations of MgCl_2_ (**a**) and LiCl (**b**) on the separation performance of TFC-BPEI, separation factor, and Mg^2+^/Li^+^ mass ratio in the permeate of the TFC-BPEI membrane with different Mg^2+^/Li^+^ mass ratios in the feed solution (**c**) and the separation performance for a 2000 mg L^−1^ MgCl_2_/LiCl mixed-salt solution at a Mg^2+^/Li^+^ mass ratio of 50:1 (**d**).

Firstly, we performed the separation test for the two kinds of membranes with salt concentrations. As shown in Figure 8a,b, TFC-BPEI has a PWP of 189.3 L m^−2^ h^−1^ MPa^−1^ with zero content of MgCl_2_ and LiCl. With increasing feed concentration, their water permeances gradually decrease for both feed solutions. The decrease in water permeance with MgCl_2_ concentration is more obvious, from 141.9 to 105 L m^−2^ h^−1^ MPa^−1^ as MgCl_2_ concentration increases from 500 to 3000 mg L^−1^. This phenomenon could be well explained by the non-equilibrium thermodynamic model (Kedem–Katchalsky model) [42,43]. Both MgCl_2_ and LiCl concentrations have little effect on their rejection, with the rejections of MgCl_2_ and LiCl always maintained at about 94% and 16%, respectively. The rejection of LiCl is lower than most of those in the literature indicates that TFC-BPEI is highly beneficial for Li^+^ penetration.

The Mg^2+^/Li^+^ selectivity of TFC-BPEI with different Mg^2+^/Li^+^ feed mass ratios were tested, as shown in Figure 8c. When the Mg^2+^/Li^+^ feed mass ratio increases above 50:1, the permeate Mg^2+^/Li^+^ mass ratio increases, and the *S*_Li/Mg_ shows a slightly downward trend. The calculated *S*_Li/Mg_ are 21.0, 22.7, 21.8, and 19.6 for Mg^2+^/Li^+^ feed mass ratios of 20:1, 50:1, 100:1, and 200:1, respectively, proving that TFC-BPEI has relatively stable Mg^2+^ and Li^+^ selective performance for mixed solutions of different concentrations.

For comparison, a Mg^2+^/Li^+^ separation test of both membranes at fixed Mg^2+^/Li^+^ mass ratios of 50:1 was conducted, as shown in Figure 8d. The feed solution contains 489 mg L^−1^ Mg^2+^ and 9.7 mg L^−1^ Li^+^. After filtration, the permeate solution of TFC-0 contains 408 mg L^−1^ Mg^2+^ and 7.8 mg L^−1^ Li^+^, and *S*_Li/Mg_ is only 0.96. Comparatively, in the permeate of TFC-BPEI, the concentration of Mg^2+^ sharply decreases to 29.4 mg L^−1^, but that of Li^+^ increases to 13.2 mg L^−1^, which is a typical Donnan exclusion effect [22]. We consider that the superior Mg^2+^/Li^+^ selective performance could be due to the special double-charged-layer structure of the membrane we fabricated. The outermost PEI modification layer is positively charged, but is a little looser due to the shortening of the interfacial polymerization time, which has been demonstrated in Section 3.1. This means it has a higher repellent force for divalent cations due to Donnan exclusion, but a lower repellent force for monovalent cations. Therefore, monovalent cations could easily penetrate through the outermost BPEI modification layer. Due to the more negatively charged carboxyl groups of the PIP/TFC polyamide layer, as shown in Figure 7b, once the monovalent cations penetrate through the BPEI modification layer, there is no longer a repellent force but an attraction force between these cations and the negatively charged carboxyl groups inside the PIP/TFC polyamide layer. The negatively charged carboxyl groups serve as a fixed charge to help the monovalent cations penetrate through the polyamide layer via ion exchange, similar to cationic ion-exchange membranes [44,45]. Therefore, this is highly beneficial for their penetration.

**Figure 9:**
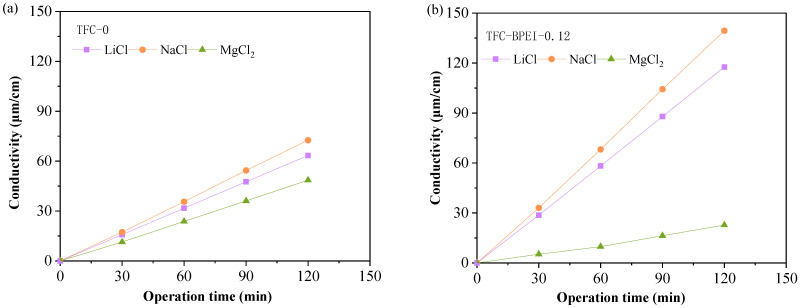

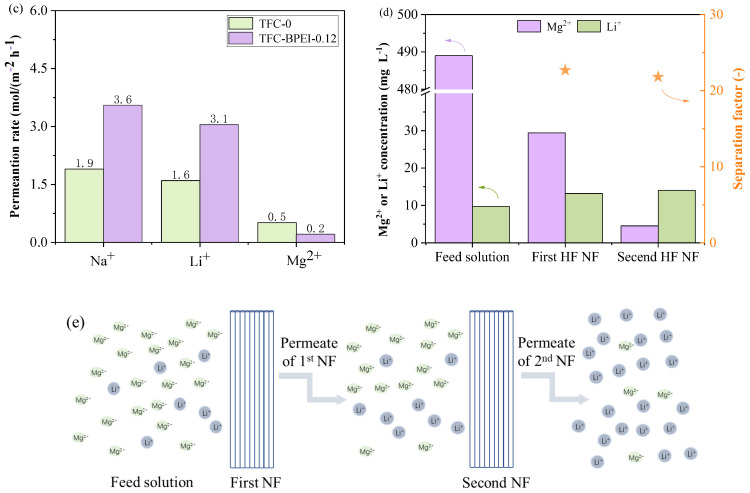
Ion permeation in the concentration-driven diffusion experiment. Change in ions’ conductivity over time in the permeate side solution through TFC-0 (**a**) and TFC-BPEI (**b**); permeation rates of ions through TFC-0 and TFC-BPEI (**c**); separation results (**d**); and the two-pass HF NF separation process for TFC-BPEI (**e**).

In order to demonstrate that the double-charged layer structure is highly beneficial for the penetration of monovalent cations and to test the selective transport ability of the two kinds of membranes for different valent cation ions, we further conducted an ion permeation experiment. We used 0.2 mol L^−1^ NaCl, LiCl, and MgCl_2_ solutions, respectively, which were circulated through the HF membrane lumens under atmospheric pressure, and we used DI water for external circulation on the outer side of the HF membrane. We measured the conductivity of the externally circulated DI water over time. The results are shown in Figure 9a,b. Based on these conductivity results, the ion permeability of the two types of membranes can be obtained, as shown in Figure 9c.

It can be seen from Figure 9c that, for TFC-0, the permeability values for Li^+^ and Na^+^ ions are quite similar to each other and are three to four times that of Mg^2+^. Theoretically, the negatively charged skin layer is beneficial for the penetration of cation ions by ionic exchange. This is the reason that common NF membranes fabricated by interfacial polymerization between PIP and TMC have poor rejection for cations, regardless of whether they are monovalent cations or divalent cations. However, Mg^2+^ has a higher molecular weight than Li^+^ or Na^+^ and the diffusivity of Mg^2+^ is smaller than those of Li^+^ and Na^+^ [46]. Meanwhile, Mg^2+^ has a slightly larger molecular diameter than Li^+^ or Na^+^. Therefore, for TFC-0, the permeability values for Li^+^ and Na^+^ are much higher than that of Mg^2+^. In comparison, from Figure 9b, we can see that, for TFC-BPEI, the permeability values for different salts have even larger differences; the permeability values for LiCl and NaCl are six to seven times that of MgCl_2_. This can be well explained by the positively charged surface, since it exerts a very high repellent force on Mg^2+^ rather than on Li^+^ and Na^+^ ions, and thus inhibits the diffusion of Mg^2+^ and results in lower permeability. Meanwhile, it is interesting that the permeability value for MgCl_2_ of TFC-BPEI is much lower than that of TFC-0, less than half of the latter. Still, this could be well explained by the surface charge properties of these two kinds of membranes. Nevertheless, it is interesting that the permeability values for LiCl and NaCl of TFC-BPEI are much higher than those of TFC-0, nearly twice that of the latter. This unusual phenomenon could be well explained by the fact that the skin layer of TFC-BPEI is thinner, which has been demonstrated in Section 3.2.2. Therefore, the diffusion resistance of the monovalent salt ions is smaller inside the skin layer of TFC-BPEI than that of TFC-0, and the permeability to monovalent salts of TFC-BPEI is greater. Meanwhile, the skin layer of TFC-BPEI is slightly looser due to the shortening of the interfacial polymerization time, which has been demonstrated in Section 3.1. Furthermore, the negatively charged polyamide layer beneath the loose positively charged BPEI layer is beneficial for the penetration of cation ions by ionic exchange [47]. Therefore, TFC-BPEI is more beneficial for the penetration of LiCl and NaCl despite its positively charged surface.

Furthermore, a secondary pass nanofiltration test on Mg^2+^/Li^+^ feed mass ratios of 50:1 was carried out. The result is shown in Figure 9d. After first-pass filtration, the permeate Mg^2+^/Li^+^ mass ratio decreased to 2.3:1. After second-pass filtration, the permeate Mg^2+^/Li^+^ mass ratio became 1:3.0, which shows a promising enrichment of Li^+^ ions. The separation factors for the two nanofiltration pass processes were 22.6 and 21.8, respectively, which are much higher than those in many state-of-the-art works. According to the results in Figure 8, the *S*_Li/Mg_ of TFC-PEI-0.12 is as high as 19.6–22.6. Clearly, TFC-BPEI is suitable for enriching Li^+^ from salt-lake brine.

### 3.4. Stability Performance

**Figure 10:**
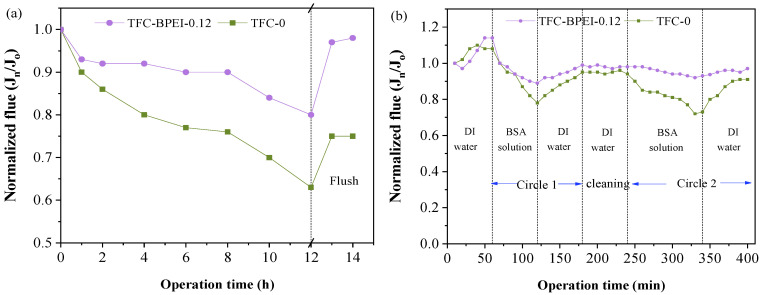

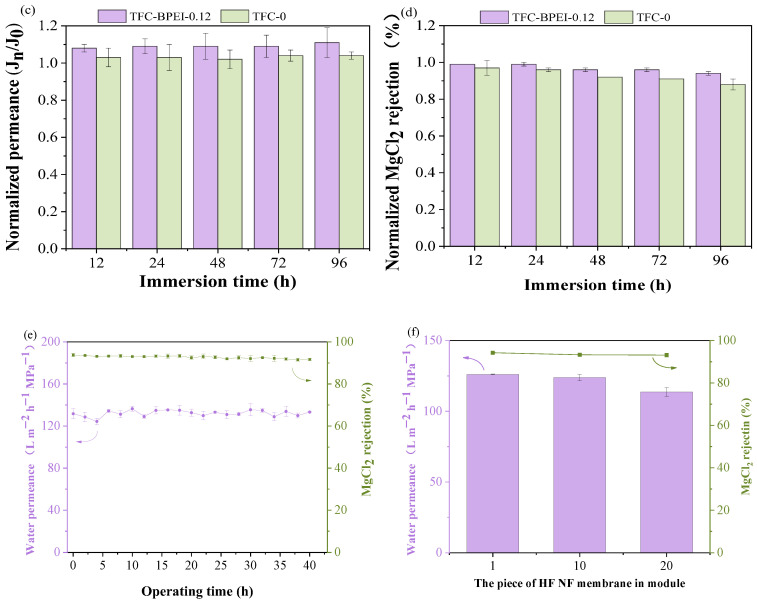
Anti-scaling performance (**a**), anti-fouling performance (**b**), and acid resistance after long-term exposure to HCl solution at pH = 3 for TFC-0 and TFC-BPEI (**c**,**d**); long-term performance of TFC-BPEI (**e**); and amplification of TFC-BPEI membranes (**f**).

During the filtration process, some weakly soluble salts such as CaCO_3_, MgCO_3_, BaSO_4_, etc. exceed their dissolution limit near the membrane surface and form scaling, thus resulting in membrane fouling and greatly decreasing the water permeability. Therefore, the scaling resistance properties of TFC-0 and TFC-BPEI were measured, as shown in Figure 10a. Both membranes have a rapidly decreased flux within the initial 1 h, which could be mainly due to a concentration polarization phenomenon that increases the mass transfer resistance [48]. However, the decrease trend for TFC-BPEI is much slower, which indicates that the concentration polarization on the TFC-BPEI surface is not severer than on TFC-0, due to the different surface charges of these two kinds of membranes. For TFC-BPEI, as its surface is positively charged, it exerts more repelling force towards divalent cation ions. Therefore, the Ca^2+^ ion concentration near the TFC-BPEI surface is surely less than that near the TFC-0 surface, as it is negatively charged and will surely attract more divalent cation ions.

With the filtration time further extended to 8 h, both membranes show different trends in normalized flux. TFC-BPEI shows only a slightly decreased normalized flux, while TFC-0 shows a remarkably decreased flux. As the filtrate is continuously discharged from the system during the filtration process due to the concentration polarization phenomenon and the high rejection of divalent cations, as well as due to the large diameter of SO_4_^2−^ ions and their low diffusion rate [12], the concentration of both Ca^2+^ ions and SO_4_^2−^ ions near the membrane surface continues to increase. When the activity product of the Ca^2+^ ions and SO_4_^2−^ ions exceeds the solubility product of CaSO_4_, crystals precipitate on the membrane surface to form CaSO_4_ precipitation. Therefore, with filtration going on, a CaSO_4_ cake layer is gradually formed, and membrane flux gradually decreases.

CaSO_4_ occurs in an induction nucleation process at this stage [49], but filter cake has not yet formed on the membrane surface, and the CaSO_4_ induction period for TFC-BPEI is longer due to its strong repelling of Ca^2+^. In contrast, the water flux of TFC-0 decreases very quickly in this filtration time range, as the membrane has a strong attracting effect on Ca^2+^, so that CaSO_4_ nucleates more on the TFC-0 surface during this induction period. 

With the filtration time extended even further from 8 h to 12 h, both membranes show an accelerated decrease in normalized flux. This indicates that CaSO_4_ begins to deposit on the membrane surface and form filter cake, and thus remarkably increases hydraulic resistance. The normalized flux of TFC-BPEI is always higher. Still, this can be well explained by the positively charged surface of TFC-BPEI, which can effectively repulse Ca^2+^, which demonstrates that it has good anti-scaling performance.

After the scaling resistance test, we flushed the two types of membranes with ultra-pure water, and the normalized flux of TFC-BPEI recovered to 97%, significantly higher than that of TFC-0 (75%). This, from one perspective, demonstrates that clean water rinsing can effectively restore the separation performance of TFC-BPEI after CaSO_4_ scaling. From another perspective, it shows that the CaSO_4_ scale formed on the surface of TFC-BPEI is surely much looser and can be easily eliminated. Therefore, the TFC-BPEI fabricated in this work is promising for the applications of water softening, magnesium–lithium separation, and in some other conditions that can form scaling on the membrane surface.

As membranes are inevitably contaminated by organic matter during practical operation, and their separation performance and service life are gradually reduced [50], we evaluated the anti-fouling resistance of TFC-0 and TFC-BPEI using 500 ppm BSA as a feed solution, and the results are shown in Figure 10b. Both TFC membranes were first conducted in deionized water for about 1 h, and flux increased during operation, which might be due to the fact that the membrane pores of the HF membrane are fully opened at a 0.4 MPa operating pressure. After that, the feed solution was changed to BSA solution to filtrate for 1 h; the water flux of both types of membrane decreased significantly with time, but the water flux of TFC-BPEI decreased less than that of TFC-0. The flux of the latter decreased to 78% of the initial amount, while that of the former decreased to 89% of its initial value, indicating that TFC-BPEI has better anti-fouling performance. The two membranes were then flushed with deionized water, and TFC-BPEI had a flux recovery rate of 98% while TFC-0 had a flux recovery rate of 94%, indicating that TFC-BPEI was more easily recovered after cleaning of the fouling substance. During the second cycle of BSA fouling testing and cleaning, the flux of TFC-BPEI decreased to 92% of its initial value and the recovery rate after cleaning was 97%, while the flux of TFC-0 decreased to 72% of its initial value and its recovery rate after cleaning was 91%. It should be pointed out that the isoelectric points of BSA range 4.6–5.8 [51], and, at neutral pH, BSA is negatively charged. In this case, the positively charged TFC-BPEI membrane had poorer anti-fouling performance than the negatively charged TFC-0 membrane. However, it showed a reverse result, which could be attributed to the much smoother surface of TFC-BPEI than TFC-0, as was demonstrated by the AFM characterization in Section 3.2, since the PEI-modified TFC-BPEI membrane is much smoother than TFC-0, making it more difficult for contaminants to adhere onto the membrane surface according to the literature [52]. Meanwhile, from the XPS characterization, we can see that there are still some negatively charged O-C=O groups on the BPEI-modified surface, which indicates that, although the nascent membrane is modified by the BPEI/ethanol solution, there is still some hydrolyzation of acyl groups. This might occur during the interfacial polymerization process or during the surface modification process, which, in turn, would also repel BSA molecules to some extent. Therefore, TFC-BPEI has better anti-fouling performance and application prospects than TFC-0.

For Li extraction from salt-lake brine, the pH of chloride-type and magnesium-sulfate-type salt-lake brine shows a gradual decreasing trend during evaporation and concentration [23,53]. The acid resistance properties of TFC-0 and TFC-BPEI were evaluated via periodic immersion in HCl solution at pH = 3 for 96 h, and the results are shown in Figure 10c,d. TFC-BPEI has slightly higher normalized water permeability compared with TFC-0. This might be due to expansion of the pores in the separation layer caused by the strong acid, as TFC-BPEI has a lower degree of crosslinking due to its much shorter interfacial polymerization time, as has been demonstrated in Section 3.2. However, the MgCl_2_ rejection of TFC-BPEI is less affected by the acidic environment than that of TFC-0, as shown in Figure 10d. This might be due to the formation of negatively charged carboxyl groups after the attack of the H^+^ ions on the vulnerable amide bonds in the separation layer, and, for TFC-BPEI, the short interfacial polymerization time results in fewer amide bonds in the separation layer that are susceptible to H^+^ attack [54,55]. Therefore, TFC-BPEI has excellent chemical stability under acidic conditions and thus could tolerate acidic-solution circumstances in magnesium–lithium separation.

The extended filtration performance of TFC-BPEI is shown in Figure 10e. During crossflow filtration of more than 40 h, MgCl_2_ rejection only decreases slightly, by 1.7%, and remains above 92%, accompanied by quite stable water permeability, remaining at around 130 L m^−2^ h^−1^ MPa^−1^, which demonstrates its good long-term operational stability and good application prospects.

We further carried out amplification of the TFC-BPEI module to contain 10 and 20 pieces of fiber, respectively, and the separation performance of these two kinds of modules were tested and compared with the module containing a single pieces of fiber, as shown in Figure 10f. The slightly decreased water permeability of the membrane module with 20 filaments might be due to a short interfacial polymerization time that results in uneven distribution of the organic phase solution on the inner surface of the hollow fibers. This is similar to the magnification results in the literature [56]. However, these three kinds of modules have about the same MgCl_2_ rejection, at around 94%, which indicates the successful amplification of the positively charged HF NF membrane module.

### 3.5. Benchmark

We compared the separation performance of the fabricated TFC-BPEI with those of other HF NF membranes in recent literature, as shown in Table 4. TFC-BPEI has a reasonably high MgCl_2_ rejection of 94.6%, a much higher PWP of 189 L m^−2^ h^−1^ MPa^−1^, and a water permeance of 126.2 L m^−2^ h^−1^ MPa^−1^; both are also relatively high compared with other positively charged HF NF membranes. It can be seen that most of the membranes in the literature are not made for Mg/Li separation, so that they did not provide LiCl rejection data or $S_{Li/Mg}$ data. However, most of them provided NaCl rejection data. Therefore, we calculated their $S_{NaCl/Mg{Cl}_{2}}$ values based on Equation (1). For TFC-BPEI, since its skin layer is much looser and thinner, the NaCl rejection of TFC-BPEI is only 12.2%, while its $S_{NaCl/Mg{Cl}_{2}}\;$is 16.6, which is among the highest in the literature. Comparatively, many membranes in the state-of-the-art literature have NaCl rejection values much higher than 20% or even than 40%, and they have lower $S_{NaCl/Mg{Cl}_{2}}$ values.

As most research works on Mg/Li separation use positively charged flat-sheet NF membranes, for comparison, we also present the separation performance of some recently reported positively charged flat-sheet NF membranes in Table 4. It can be seen that most of them have much higher MgCl_2_ rejection values and reasonable water permeance compared with positively charged hollow-fiber NF membranes. Nevertheless, most of the former have relatively low $S_{Li/Mg}$, below 15, while the $S_{Li,Mg}\;$of TFC-BPEI is 22.7. Therefore, TFC-BPEI has an excellent ability to separate monovalent and divalent cation ions compared with both hollow-fiber and flat-sheet NF membranes and shows broad industrial application prospects in the field of magnesium–lithium separation.

## 4. Conclusions

A double-charged layer structure was formed by surface modification using a BPEI/ethanol solution to react with residual -COCl groups on the pristine HF NF membrane surface. The as-prepared HF membrane has excellent selective separation performance for divalent cations due to the combined effect of Donnan exclusion and steric hindrance, while it can promote more monovalent cations to penetrate through due to its relatively looser surface and the negative charged nature of the polyamide layer beneath its outermost positively charged BPEI layer. In this way, the BPEI-modified TFC HF NF membrane shows a typical characteristic of positively charged membranes and achieves an excellent selectivity (*S*_Li/Mg_) of more than 20 despite the different Mg^2+^/Li^+^ mass ratios used, which is among the highest for HF NF membranes with positively charged surfaces in recent literature. Further, the HF NF membrane has a much smoother morphology and greatly increased hydrophilicity, which leads to superior anti-scaling and anti-fouling performance, and it can be more easily recovered after cleaning of the scaling and fouling substance. The positively charged HF NF membrane has excellent chemical stability under acidic conditions as well. Furthermore, it has been successfully amplified and still retains good separation performance. Therefore, the positively charged HF NF membrane is suitable for mono/divalent cation separation, especially Li^+^ enrichment from salt-lake brine, as well as water softening.

## Figures and Tables

**Table 1:** Modification conditions of the HF NF membranes.

Membrane	Modification Solution (wt%)
TFC-BPEI-0.04	BPEI/EtOH: 0.04/99.96
TFC-BPEI-0.08	BPEI/EtOH: 0.08/99.92
TFC-BPEI-0.12	BPEI/EtOH: 0.12/99.88

**Table 2:** Contents of the chemical groups of TFC HF NF and TFC-BPEI membranes analyzed from the XPS fitting.

Element	Components	TFC-0	TFC-BPEI
		Composition (%)	Composition (%)
N	-N-C	97.6	82.9
	>N^+^H_2_/-N^+^H_3_	2.4	4.4
	R-NH_2_	0	12.7
O	-C=O	67.9	83.8
	-OH	32.1	16.2

**Table 3:** Contents of the chemical groups in TFC-BPEI-EtOH-5nm, TFC-BPEI-EtOH-15nm, and TFC-BPEI-Water-5nm membranes analyzed from the XPS fitting.

Element	Components	TFC-BPEI-EtOH-5nm		TFC-BPEI-EtOH-15nm		TFC-BPEI-Water-5nm	
		Composition (%)		Composition (%)		Composition (%)	
O	-OH	19.6		23.3		32.5	
	-C=O	80.4		76.7		67.5	
N	-N-C	82.6		86.7		86.7	
	>N^+^H_2_/-N^+^H_3_	2.2	17.4	1.2	13.3	7.5	13.2
	R-NH_2_	15.2		12.1		5.7	

**Table 4:** Comparison of performance between TFC-BPEI and other positively charged hollow-fiber membranes and some of the flat-sheet NF membranes in recent literature.

Membrane	Type	LiCl/NaCl Rejection %	MgCl_2_ Rejection %	$S_{LiCl/Mg{Cl}_{2}}$ $/S_{Li/Mg}$ $/S_{NaCl/Mg{Cl}_{2}}$	Water Permeance (L m^−2^ h^−1^ MPa^−1^)	Ref.
PIP/TMC/BPEI	HF	15.6/12.2	94.7	15.9/22.7/16.6	125.7 189.3 (PWP)	This work
(PIP+BPEI)/TMC/TAEA	HF	—/61.6	97.6	—/—/16.0	160.0 (PWP)	[27]
PEI/TMC	HF	—/48.0	97.0	—/—/17.3	88.3	[57]
(PIP+BPEI)/TMC	HF	—/53.6	96.3	—/—/12.5	182.0 (PWP)	[17]
PAI/PAAm	HF	—/11.7	95.7	—/—/20.5	156.0	[58]
cL/TMC	HF	—/27.6	95.1	—/—/14.8	103.0	[11]
HBPEI/GA	HF	—/84.1	94.2	—/—/2.7	94.0	[59]
PVA/PQ-10/GA	HF	—/35.0	86.9	—/—/5.0	85.0	[60]
DAPP/TMC	HF	21.8/23.0	71.4	2.7/—/2.7	26.3	[40]
SWCNT/PIP/TMC/PEI	FS	46.2/—	98.5	35.8/33.4/—	74.0	[61]
PEI/TMC/DAIB	FS	55.6/—	95.8	10.6/—/—	132.0	[62]
PEI/TMC/QEDTP	FS	55.8/61.3	95.6	10.0/15.6/8.8	112.9	[63]
PILNH_2_/TMC	FS	32.6/—	95.0	13.4/10.3/—	116.7	[19]
(PEI+Cyclen)/TMC	FS	22.0/—	90.4	8.1/8.7/—	140.0	[15]

## Data Availability

The data are contained within the article and Supplementary Material.

## Supplementary Materials

The following supporting information can be downloaded at: https://www.mdpi.com/article/10.3390/membranes14010001/s1, Figure S1. ATR-FTIR spectra of the PSf substrate, TFC-0 HF NF, and TFC-BPE HF NF membranes. Figure S2. ATR-FTIR spectra of TFC-BPEI-0.12-2, TFC-BPEI-0.04-2, and TFC-BPEI-0.12-1 membranes. Figure S3. Schematic diagram of ion permeation: experimental setup for concentration-driven diffusion. Figure S4. Wide-scan XPS spectra of the TFC-0 and TFC-BPEI-0.12-2 membranes. Table S1. Spinning parameters of PSf hollow-fiber substrate membranes. Table S2. Atomic percentages of the TFC-0 and TFC-BPEI-0.12-2 membranes. Refs. [64,65,66,67,68,69] are cited in the Supplementary Materials.
