# Supplementary material for: A Selective Separation Mechanism for Mono/divalent Cations and Properties of a Hollow-Fiber Composite Nanofiltration Membrane Having a Positively Charged Surface
Source: Membranes (Basel). 2023 Dec 20;14(1):1. doi: 10.3390/membranes14010001 (PMC10818550; doi:10.3390/membranes14010001)
Supplement: Supplementary file 1 [file membranes-14-00001-s001.zip › membranes-2765348-supplementary.pdf]

Supplementary

A Selective Separation Mechanism for Mono/divalent Cations and Properties of a Hollow-Fiber Composite Nanofiltration Membrane Having a Positively Charged Surface

S1. Spinning parameters of PSf hollow fiber substrate membranes

Table S1. Spinning parameters of PSf hollow fiber substrate membranes.

Spinning parameter	Value
Composition of dope solution (PSf/PVP/DMF, wt %)	20.0/2.0/78.0
Bore composition	H_2O
Bore flowrate (mL min^{-1})	0.9
Dope flowrate (mL min^{-1})	2.0
Air gap (cm)	6.0±0.2
Take-up velocity	Free fall
Outer coagulation bath	H_2O
Dope temperature (°C)	25±3
Bore temperature (°C)	25±3
Outer coagulation bath temperature (°C)	20±3
Flushing bath temperature (°C)	20±3
Environmental humidity (%)	50±5
Environmental temperature (°C)	25±3
Spinet dimension (mm)	o.d./i.d.(1.3/0.7)

S2. Characterization methods

The surface chemistry of the fabricated HF substrate and HF NF membranes were characterized by X-ray photoelectron spectroscopy (XPS, ESCALAB 250 XI, USA). The hydrophilicity of the membrane surface was characterized via a contact angle meter (CA, DSA100, Kruss, Germany) using the sessile drop method. The surface morphology of the HF NF membranes with gold sputtering was observed by scanning electron microscopy (SEM, S4800SEM, Hitachi, Japan). The surface roughness of the resultant HF NF membranes was detected by atom force microscopy (AFM, Agilent 5400, USA), using a scanning scope of 5.0 * 5.0 μm^2, and the result is expressed as root-mean-square roughness (*S*q, nm).

S3. ATR-FTIR characterization

The infrared spectral results of the PSf HF UF membrane, TFC-0 HF NF membrane and TFC-BPEI HF NF membrane are shown in Error! Reference source not found.**1**. All the three membranes show typical characteristic peaks at 1505, 1240 and 1150 cm^{-1}, which belong to the C-H symmetric deformation vibration peak of $C(CH_3)_2$ of PSf, the asymmetric telescopic vibration peak of C-O-C in the aryl (Ar)-O-Ar group, and the symmetric telescopic vibration peak of sulfonyl group (O=S=O) [1, 2]. both the TFC-0 and TFC-BPEI-0.12-2 membrane had typical characteristic peaks at 1632 cm^{-1}, which was attributed to the C=O tensile vibration in the amide bond, which could prove the successful construction of polyamide layer on the inner surface of the HF substrate. The PSf HF UF membrane has a distinct characteristic peak at 1662 cm^{-1}, which is mainly attributed to the tensile vibration of C=O by a small amount of the porous agent PVP [3]. The peak of TFC-0 and TFC-BPEI-0.12-2 membrane around 1680 cm^{-1} is the C=O tensile vibration of the amide group [4].

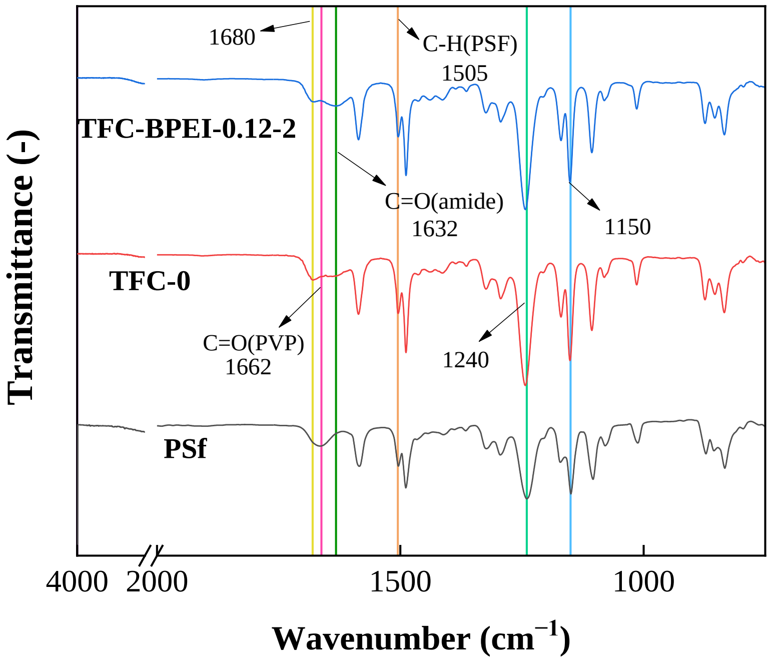

Figure S1. ATR-FTIR spectra of PSf substrate, TFC-0 HF NF and TFC-BPE HF NF membranes.

In order to more intuitively see the influence of different BPEI modification conditions on the chemical composition and structure of the inner surface of the membrane, we prepared TFC-BPEI-0.04-2 and TFC-BPEI-0.12-1 membrane, and characterized them by infrared spectroscopy. The characterization results are shown in Error! Reference source not found.**2**. The TFC-BPEI HF NF membrane prepared under different modified conditions only had a characteristic peak difference near the C=O telescopic vibration peak of the amide bond, that is, 1632 cm^{-1}, among which the TFC-BPEI-0.12-2 membrane had a more obvious peak at this position, which indicates that the reaction between BPEI and the remaining acid chloride groups in the interfacial polymerization under this condition is more completely and thus generates more amide groups.

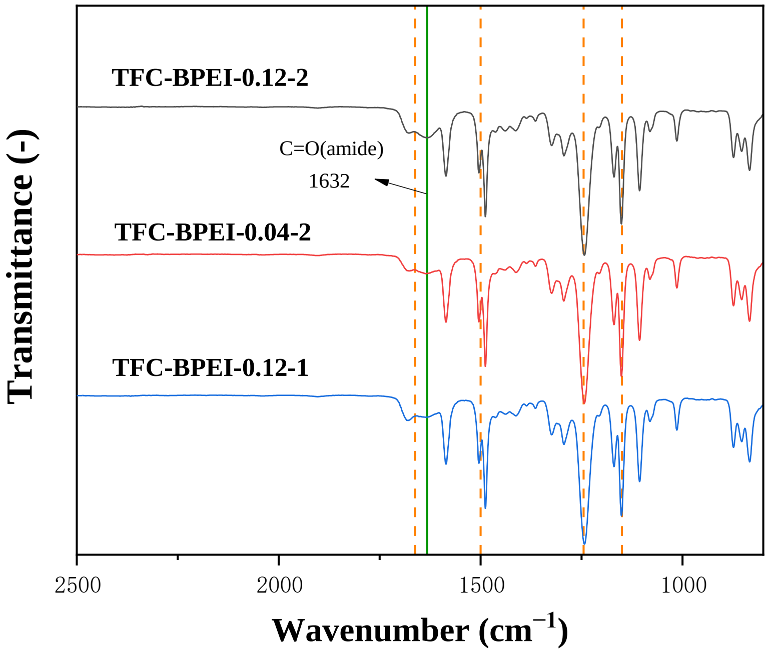

Figure S2. ATR-FTIR spectra of TFC-BPEI-0.12-2, TFC-BPEI-0.04-2 and TFC-BPEI-0.12-1 membranes.

S4. The ions permeation experiment of concentration-driven diffusion

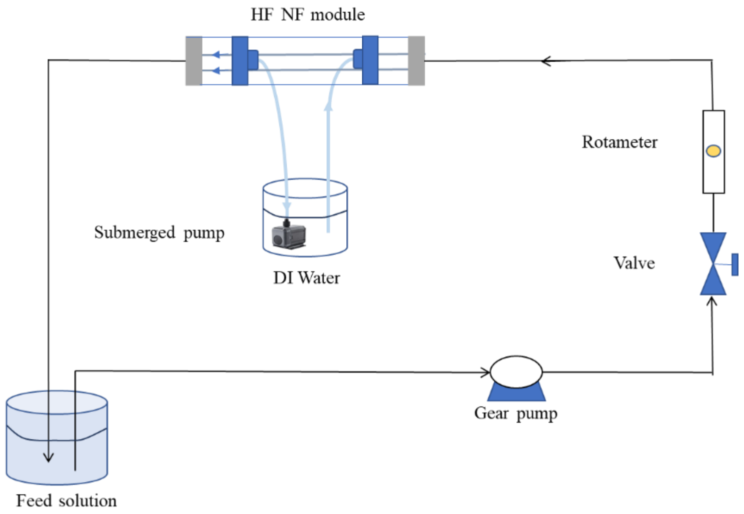

Figure S3. Schematic diagram of ions permeation experimental setup of concentration-driven diffusion.

S5. XPS characterization

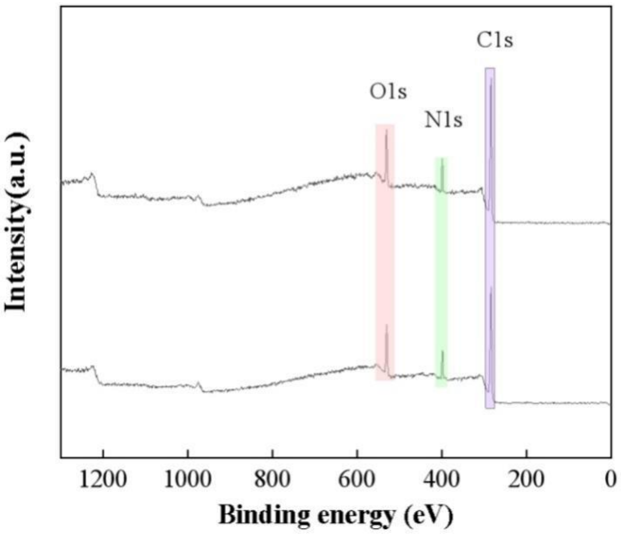

Figure S4. Wide scan XPS spectra of TFC-0 and TFC-BPEI-0.12-2 membranes.

Table S2. Atomic percentage of the TFC-0 and TFC-BPEI-0.12-2 membranes.

Membrane	Atomic percent (at%)		
	C	N	O
TFC-0	78.15	9.36	12.41
TFC-BPEI-0.12-2	81.02	9.11	9.79

As shown in **Table S2**, the oxygen content of TFC-BPEI-0.12-2 membrane is significantly lower than that of TFC-0 membrane, which is due to the secondary interfacial reaction between BPEI and the residual unreacted acid chloride group on the surface of the polyamide layer after the interface polymerization, so as to avoid the hydrolysis of these acid chloride group, which reduces the oxygen content on the surface of the membrane. At the same time, the C content in the separation layer increased and the N and O contents decreased, which indicated that the separation layer contained a large amount of BPEI, which proved the success of the modification, that is, the separation layer had a high degree of amino grafting.

References

1. Jiang, Y.; Li, S.; Su, J.; Lv, X.; Liu, S.; Su, B. Two dimensional COFs as ultra-thin interlayer to build TFN hollow fiber nanofiltration membrane for desalination and heavy metal wastewater treatment. *J. Membr. Sci.* **2021**, *635*, 119523.
2. Yu, H.; Cao, Y.; Kang, G.; Liu, J.; Li, M.; Yuan, Q. Enhancing antifouling property of polysulfone ultrafiltration membrane by grafting zwitterionic copolymer via UV-initiated polymerization. *J. Membr. Sci.* **2009**, *342*, 6–13.
3. Lv, X.; Wang, E.; Liu, S.; Liu, L.; Yin, Y.; Li, S.; Su, B.; Han, L. Tannic acid reinforced interfacial polymerization fabrication of internally pressurized thin-film composite hollow fiber reverse osmosis membranes with high performance. *Desalination* **2022**, *538*, 115926.
4. Li, C.; Li, S.; Zhang, J.; Yang, C.; Su, B.; Han, L.; Gao, X. Emerging sandwich-like reverse osmosis membrane with interfacial assembled covalent organic frameworks interlayer for highly-efficient desalination. *J. Membr. Sci.* **2020**, *604*, 118065.
5. Kedem, O.; Katchalsky, A. Thermodynamics analysis of the permeabilityof biological membranes to non-electrolytes. *Biochim. Biophys. Acta* **1958**, *27*, 229–246.
6. Baranowski, B. Nan-equlitbrun thermodynarnics as appied to membrane ransport. *J. Mermbrane Sci.* **1991**, *57*, 119–159.
7. Feng, C.; Xu, J.; Li, M.; Tang, Y.; Gao, C. Studies on a novel nanofiltration membrane prepared by cross-linking of polyethyleneimine on polyacrylonitrile substrate. *J. Membr. Sci.* **2014**, *451*, 103–110.
8. Diaz, P.A.B.; Kronemberger, F.D.A.; Habert, A.C. Effect of feed conditions and added solutes on the performance of membrane nanofiltration of succinic acid solutions. *Braz. J. Chem. Eng.* **2020**, *37*, 283–295.

9. Ward, L.M.; Fickling, B.G.; Weinman, S.T. Effect of Nanopatterning on Concentration Polarization during Nanofiltration. *Membranes* **2021**, *11*, 961.

Disclaimer/Publisher's Note: The statements, opinions and data contained in all publications are solely those of the individual author(s) and contributor(s) and not of MDPI and/or the editor(s). MDPI and/or the editor(s) disclaim responsibility for any injury to people or property resulting from any ideas, methods, instructions or products referred to in the content.
